# Ancestral Sequence Reconstructions of MotB Are Proton-Motile and Require MotA for Motility

**DOI:** 10.3389/fmicb.2020.625837

**Published:** 2020-12-23

**Authors:** Md Imtiazul Islam, Angela Lin, Yu-Wen Lai, Nicholas J. Matzke, Matthew A. B. Baker

**Affiliations:** ^1^School of Biotechnology and Biomolecular Sciences (BABS), University of New South Wales, Sydney, NSW, Australia; ^2^School of Biological Sciences, University of Auckland, Auckland, New Zealand; ^3^CSIRO Synthetic Biology Future Science Platform, Brisbane, QLD, Australia

**Keywords:** motility, flagellar and chemotaxis, stator, ancestral sequence reconstruction, ion-selectivity

## Abstract

The bacterial flagellar motor (BFM) is a nanomachine that rotates the flagellum to propel many known bacteria. The BFM is powered by ion transit across the cell membrane through the stator complex, a membrane protein. Different bacteria use various ions to run their BFM, but the majority of BFMs are powered by either proton (H^+^) or sodium (Na^+^) ions. The transmembrane (TM) domain of the B-subunit of the stator complex is crucial for ion selectivity, as it forms the ion channel in complex with TM3 and TM4 of the A-subunit. In this study, we reconstructed and engineered thirteen ancestral sequences of the stator B-subunit to evaluate the functional properties and ionic power source of the stator proteins at reconstruction nodes to evaluate the potential of ancestral sequence reconstruction (ASR) methods for stator engineering and to test specific motifs previously hypothesized to be involved in ion-selectivity. We found that all thirteen of our reconstructed ancient B-subunit proteins could assemble into functional stator complexes in combination with the contemporary *Escherichia coli* MotA-subunit to restore motility in stator deleted *E. coli* strains. The flagellar rotation of the thirteen ancestral MotBs was found to be Na^+^ independent which suggested that the F30/Y30 residue was not significantly correlated with sodium/proton phenotype, in contrast to what we had reported previously. Additionally, four among the thirteen reconstructed B-subunits were compatible with the A-subunit of *Aquifex aeolicus* and able to function in a sodium-independent manner. Overall, this work demonstrates the use of ancestral reconstruction to generate novel stators and quantify which residues are correlated with which ionic power source.

## Introduction

Bacterial cells can move through liquids or over moist surfaces using rotating flagella to propel themselves in response to chemical stimulus, temperature and pH (Armitage, 2007; Jarrell and McBride, 2008; Gurung et al., 2020). The bacterial flagellar motor (BFM) drives the rotation of the flagellum (Sowa and Berry, 2008). One of the largest molecular machines in bacteria, with a molecular mass of ∼11 MDa (Sowa and Berry, 2008), the BFM is present in a great variety of bacterial taxa from various habitats (Pion et al., 2013). It shares structural and amino acid sequence homology across a diverse range of taxa which suggests early ancestry and makes it a case study for investigating the origins of microbial motility (Mitchell and Kogure, 2006; Thormann and Paulick, 2010; Son et al., 2015).

The stator complexes are motor-associated protein complexes which form a selective ion channel that converts chemical energy into mechanical torque to rotate the rotor (Minamino et al., 2018). The stator proteins are divided into A- and B-subunits and named according to the transferred ion, for example, MotA and MotB in H^+^ powered motors in *Escherichia coli*, and PomA and PomB in Na^+^ powered motors in *Vibrio alginolyticus* (Yorimitsu and Homma, 2001; Berg, 2003). According to the complete structure of stator complex, the A-subunit (MotA/PomA) consists of four transmembrane (TM) domains and a large cytoplasmic domain that is proposed to interact with the rotor through the rotor component FliG and generate torque (Dean et al., 1984; Blair and Berg, 1991; Zhou et al., 1995; Braun et al., 2004; Onoue et al., 2019). The B-subunit (MotB/PomB) contains a single TM domain followed by a large periplasmic domain which consists of a plug segment to control ion flow and a peptidoglycan-binding domain (PGD) to interact with peptidoglycan layer (Roujeinikova, 2008; Kojima et al., 2018). The stoichiometry of the stator units was until recently believed to be 4(A):2(B) according to the available previous crosslinking, biochemical, and genetic data of MotAB and PomAB (Sato and Homma, 2000; Kojima and Blair, 2001; Kojima et al., 2009). However, in 2020 the full structure of the stator complex was solved and it was reported that the stoichiometry of the stator unit is 5:2 instead of 4:2 for both MotAB/PomAB families (Deme et al., 2020; Santiveri et al., 2020). The new reports suggested that five copies of MotA enclosed the two TM domains from of the two copies of MotB (Deme et al., 2020; Santiveri et al., 2020). The four TM domains of MotA are arranged in two layers, TM3 and TM4 line the central pore, while TM1 and TM2 form a surrounding outer layer of helices (Deme et al., 2020; Santiveri et al., 2020). TM1 and TM2 stabilize the assembly of MotA whereas TM3 and TM4 make direct interactions with MotB, and both of them together span the complete height of MotA and extend to the cytoplasmic domain (Deme et al., 2020; Santiveri et al., 2020). The functional mechanism of MotAB starts with the dimerization and binding of PGDs to the peptidoglycan layer which leads the unplugging of the ion channel to allow ion exchange. Finally, the binding and release of proton or hydronium ions by the universally conserved MotB aspartate residue allow the MotA to bind the neighboring FliG, which trigger the conformational change in the stator complex and generate torque (Deme et al., 2020; Santiveri et al., 2020).

Ion selectivity provides flexibility to bacteria for using suitable ions according to their environmental conditions (Terahara et al., 2008). Generally, most of the bacterial species use a single stator complex to couple with specific ions such as protons (H^+^), sodium (Na^+^), potassium (K^+^), rubidium (Rb^+^), magnesium (Mg^2+^), calcium (Ca^2+^), or strontium (Sr^2+^) to drive their motor (Li et al., 2011; Terahara et al., 2012; Minamino and Imada, 2015; Imazawa et al., 2016; Ito and Takahashi, 2017). However, some bacterial species can power their flagella by coupling more than one ion using either multiple types of stator complexes or single dual-functional complexes (Terahara et al., 2006, 2008; Paulick et al., 2015). Previous studies suggested that the TM domain of the B-subunit (MotB, MotS, and PomB) of the stator complex is specifically crucial for the selectivity of H^+^ and Na^+^ ions in the flagellar motor (Asai et al., 2000; Ito et al., 2005). In the MotAB complex, a conserved aspartic acid residue (D32) at the N-terminal side of the TM region is thought to function as a universally conserved site for ion binding (Zhou et al., 1998). Mutational studies suggested that valine (V42) and leucine (L42) located at the ten amino acids downstream from the universal conserved aspartic acid (D32) are critical for the selection of H^+^ and Na^+^ ions, respectively (Terahara et al., 2008). Another study has exhibited that the methionine residue (M33) at the TM region of MotS is critical for K^+^ selectivity (Terahara et al., 2012). It has also been reported that switching of ion selectivity and use of dual ion into a single stator can be introduced with mutations and hybridizations of the ion-binding transmembrane region of the B-subunit (Terahara et al., 2008; Nishino et al., 2015). However, recently it was proposed that the MotP subunit of the MotPS complex was important for the K^+^ selectivity of the flagellar stators of *Bacillus alcalophilus* and *Bacillus trypoxylicola* (Naganawa and Ito, 2020).

Although the recent high-resolution cryoelectron microscopy reconstructions of stator units enable us to gain more detailed information of the structural and functional components of BFM that are involved in the conversion of electrochemical energy to mechanical torque (Deme et al., 2020; Santiveri et al., 2020), the exact mechanism and structural determinants of ion selectivity are yet to be discovered. However, with the help of statistical phylogenetic methods, it is possible to infer the sequences of extinct ancestral proteins from the sequences of their extant descendants (Hochberg and Thornton, 2017). It is possible to then resurrect the ancestral proteins corresponding to these inferred sequences and characterize their *in vitro* biological and biochemical properties (Gaucher et al., 2003; Thornton et al., 2003). Furthermore, ancient proteins can be expressed in contemporary hosts to examine the function of ancient proteins *in vivo*, and their integration and subsequent adaptation in ancient-modern hybrids (Kaçar and Gaucher, 2012; Kacar et al., 2017a,b). It is also possible to reconstruct the ancestral protein using the inferred ancestral sequence and express the reconstructed ancestral proteins using genetic engineering techniques to characterize their biological and biochemical properties (Gaucher et al., 2003; Thornton et al., 2003). In this study, we have built a phylogeny of MotB proteins in proteobacteria and computationally reconstructed the ancestral stator units focusing on the TM region of the B-subunit of the stator complex. We engineered selected nodes into a chimeric plasmid to characterize their motility and evaluate their ion selectivity to interrogate ion selectivity in ancient-modern hybrids of the flagellar motor at specific points in our estimate of history.

## Materials and Methods

### Phylogenetic Analysis and Ancestral Sequence Reconstruction

#### Sequence Gathering

A sequence similarity network (SSN) was generated as previously described (Atkinson et al., 2009). The protein sequences of 2187 bacterial homologs of MotB (UniProt: P0AF06) were selected using the EFI-EST server (Gerlt et al., 2015) with *E*-value = 10 for the all-versus-all BLAST, and using representative nodes to represent clusters with 90% sequence similarity. A subset of 757 sequences was selected as follows: nodes connected by edges >85% sequence identity were kept (1289 nodes, 5870 edges), and then the 405 least connected nodes (duplexes and triplexes) were removed (down to 884 nodes, 5572 edges). Sequences for these 884 nodes were aligned with MUSCLE (Edgar, 2004) and nodes were restricted to sequences that contained either FAD, YAD, or LAD at residues 30–32 (*E. coli* MotB numbering, 757 nodes, 4326 edges), to ensure conserved residue D32 for function. The clustering of characterized proteins were visualized using the gamma-organic layout on Cytoscape 3.1 (Akiva et al., 2017) and presented in Supplementary Figure 1.

### Phylogenetics

757 MotB sequences from above were aligned using Clustal Omega (Larkin et al., 2007) with five iterative re-alignments. The phylogeny was then estimated with Quicktree using Kimura translation for pairwise distance and calculating bootstraps with 100 iterations (Howe et al., 2002). The phylogeny was midpoint-rooted in FigTree (Rambaut, 2014) for display purposes, and should be treated cautiously as with any single-protein estimate of a deep phylogeny (Pallen and Matzke, 2006; Abby and Rocha, 2012; Shih and Matzke, 2013; Prangishvili et al., 2017; Ishida et al., 2019). For the purpose of surveying sequence diversity across MotBs, the tree is adequate, and arbitrary re-rooting will not affect estimation of ASRs at nodes.

### Node Selection

Thirteen nodes were selected at assessed divergent points between clades based on known sodium swimmers and their proximity and distance from the arbitrary root. Sequences (Supplementary Figure 2A) and conservation via sequence logo (Crooks et al., 2004) are shown in Supplementary Figure 2.

### Reconstruction

Ancestral protein sequences were reconstructed using the empirical Bayes method implemented in PAML (Yang, 2007). MotB sequences were truncated to focus on the TM region between residues 23–65 on MotB. The posterior probability distribution at each site for each ancestral node was also calculated using PAML.

### Bacterial Strains and Plasmids and Growth Conditions

The bacterial strains and plasmids used in this study are shown in full in Supplementary Table 1. The primary strains used in this work are RP6894 (Block et al., 1989) and RP3087 (Blair et al., 1991). All the *E. coli* strains were cultured in LB broth and LB agar [1% (w/v) Bacto tryptone, 0.5% (w/v) Bacto yeast extract, 0.5% (w/v) NaCl, and 2% (w/v) Bacto agar for solid media] at 37°C. According to the selective antibiotic resistance pattern of the plasmids, chloramphenicol (CAM), ampicillin (AMP) or kanamycin (KAN) were added to a final concentration of 25 μg/mL, 50 μg/mL, and 25 μg/mL, respectively.

### Cloning of Selected MotB-ASR Sequences

We engineered the ancestral reconstructed sequences (ASR) in a predesigned form of genetic structure that included the first 22 residues of *E. coli* MotB, followed by 42 residues (23–65) of ancestral sequence and a final 243 residues (66–308) of the MotB chassis (ASR = 1–22 MotB; 23–65 ASR; 66–308 MotB). In summary, this used the *E. coli* N-terminal domain, the ASR for the TM and plug domains (TM: residues 28–49, plug: 52–65) and then the *E. coli* PGD and C-terminal domain (PGD: 196–225). MotB-ASRs were constructed by PCR amplification using the chimeric plasmid pSHU1234 (Nishino et al., 2015) containing PomA and PotB (a hybrid of PomB and MotB) as the cloning vector. Thirteen forward ultramer primers were designed with ∼200 nucleotide overhangs that contained a *Nde*I restriction site, the predicted ancestral sequences and an annealing sequence specific to MotB on PotB in pSHU1234. A common reverse primer was designed to amplify from the end of PotB with a *Pst*I restriction site. IDT synthesized all the primers. The list of all primers is provided in Supplementary Table 2. Phusion high-fidelity (HF) DNA polymerase (NEB) was used for the PCR amplification of ASR sequences. Each reaction contained 10 μL of 5X Phusion HF buffer, 200 μM dNTPs, 0.1 μM of each primer, 10 ng of plasmid template, 3% of DMSO, 1 U Phusion HF DNA polymerase, sterile Milli-Q water to final volume of 50 μL. The reactions were started by heating to 98°C for 30 s. The PCR reactions were then subjected to 35 cycles of 98°C for 10 s, 58°C for 30 s, and 72°C for 30 s, with a final 5 min extension step at 72°C.

The amplified MotB-ASR sequences were then separated in 1% (w/v) agarose gel and purified using the Qiagen gel purification kit. Purified MotB-ASR sequences and pSHU1234 were digested with *Nde*I and *Pst*I before ligation with T4 ligase (NEB) using a 1:3 vector to insert molar ratio. All the resulting MotB-ASR plasmids contain a PomA from the vector backbone and an ancestral-contemporary MotB hybrid (Supplementary Table 1).

Finally, all the MotB-ASR plasmids were transformed into stator deleted (RP6894) and MotB deleted (RP3087) *E. coli* strains following the chemical transformation protocol from NEB. All engineered and cloning of MotB-ASR sequences were confirmed by colony PCR and Sanger sequencing at The Ramaciotti Centre for Genomics (UNSW, Australia) using ASR sequence-specific primers (Supplementary Table 2).

### Construction of Single Stator Plasmids

We constructed pMotB via deleting MotA from the MotAB plasmid pDB108 (A gift from David F Blair Lab) and constructed pPomA and pPotB by deleting PotB and PomA, respectively, from PomAPotB plasmid pSHU1234 (Nishino et al., 2015). Site-directed, Ligase-Independent Mutagenesis (SLIM) was used (Chiu et al., 2008) with specific primers (Supplementary Table 2). We also constructed a A225D point mutant of *A. aeolicus* MotA from the wild type *A. aeolicus* MotA plasmid (pNT7) (Takekawa et al., 2015) using QuikChange Lightning Site-Directed Mutagenesis with the recommended protocol from Agilent. The primers used for this point mutation were provided in the Supplementary Table 2.

### Evaluation of Swimming Properties of MotB-ASRs in Soft Agar Motility Assay

We performed swim plate motility assays according to the previous protocol (Ishida et al., 2019) with slight modifications to check whether ancestral MotB proteins could restore motility. LB swim plates [0.25% (w/v) Bacto agar] were used to screen bacteria with swimming properties. Minimal medium swim plates [10 mM of KHPO_4_, 0.1% (v/v) of glycerol, 0.1 mM of Thr, 0.1 mM of Leu, 0.1 mM of His, 0.1 mM of Met, 0.1 mM of Ser, 1 mM of MgSO_4_, 1 mM of (NH_2_)_2_SO_4_, 1 μg/mL of thiamine, and 85 mM of NaCl or 85 mM of KCl, respectively, 0.25% (w/v) agar, 0.02% (w/v) of arabinose, pH 7.0] were used to determine the dependency of coupling ions (Na^+^ or H^+^) as the medium contained only the desired ion and the pure forms of nutrients that did not contain any other ionic contaminants. Antibiotics (25 μg/mL CAM or 50 μg/mL AMP or 25 μg/mL KAN) were added according to the plasmids used. Additionally, minimal swim plates with different Na^+^ concentrations in combination with 100 μM phenamil (Sigma-Aldrich) were used to assess the of Na^+^ dependency on swimming properties.

Swim plates were inoculated with a single 1 day old colony with a sterile toothpick and incubated at 30°C for 14 h (for LB swim plates) or 16 h (minimal swim plates) to allow proper development of a swimming ring. Swimming zones were first visually checked, imaged with the ChemiDoc MP Imaging System (Bio-Rad), and swimming diameters measured using ImageJ software (Version 1.52).

### Determination of the Effect of Sodium (Na^+^) Concentration on the Rotation Speed of MotB-ASRs

The effect of different Na^+^ concentrations on the rotation speed of MotB-ASRs and sodium/proton controls were determined by tethered cell assays in the presence of a range of Na^+^ concentrations following a previous protocol (Nishiyama and Kojima, 2012) with some modifications. *E. coli* RP3087 containing MotB-ASR plasmids were inoculated into TB broth [1% (w/v) Bacto tryptone, 0.5% (w/v) NaCl] containing 0.02% (w/v) arabinose and 25 μg/ml chloramphenicol and were grown overnight (17 h) with 180 rpm at 30°C. The overnight cultures were sub-cultured with a 50-fold dilution into fresh TB broth and incubated for 5 h with 180 rpm at 30°C to get more motile cells. At OD_600_ ∼0.80, the flagella of the cells were sheared by passing the culture multiple times (∼35) through a 26G needle syringe. After shearing the flagella, the cells were washed three times with motility buffer [10 mM potassium-phosphate, 10 mM lactic acid, 100 mM NaCl, and 0.1 mM EDTA, pH 7.0]. Cells were then attached on glass slides pre-treated with an anti-*E. coli* flagellin antibody with a 1:10 dilution (Nishiyama and Kojima, 2012) and washed sequentially with a Na^+^ concentration gradient containing motility buffer. The rotational speed of the cells was observed using phase-contrast microscopy (Nikon) and was recorded at 20 frames per second (FPS) through the 40X objective with a camera (Chameleon3 CM3, Point Grey Research). Rotational motion of the cells was analyzed using Lab view 2019 software (National Instruments) and rotational speeds were calculated from 20 individual cells.

### Growth Curve Assays

Growth assays were executed to exclude possible growth impact of MotB-ASRs. Growth of *E. coli* strain RP3087 transformed with all the MotB-ASRs, pSHU1234, and pMotB plasmids were monitored as described (Santiveri et al., 2020) with slight modifications. Briefly, overnight cultures of the test strains with OD_600_ 1.0 were diluted 1:100 in 96-well plates (Corning) with fresh LB broth containing different concentrations of NaCl, 0.02% (w/v) arabinose and 25 μg/ml chloramphenicol and incubated at 37°C. The OD_600_ were measured in a microplate reader (FLUOstar OPTIMA, BMB LABTECH) every hour for 8 h with a brief shaking interval before each measurement. The experiment was performed in a triplicate.

### Correlation Analysis of Mutations at Each Respective Site of MotB Ancestral Sequences

To determine the correlation of mutations at each respective site of MotB ancestral sequences, we selected 19 species of bacteria (Supplementary Table 3) where the ion selectivity correlation with residue was previously measured (Ishida et al., 2019). Each species in the 19-species subset was classified as Na^+^ or H^+^ powered, we then added our functional MotB-ASRs as a further 13 H^+^ powered units and conducted Fisher’s Exact Test analysis (in R) based on a binary response variable. We were interested in whether a binary response might be correlated with a binary amino acid predictor, so we filtered columns in the alignment to exclude columns where the two most common amino acids added up to <85% of the observed residues. We ran Fisher’s exact test to correlate each amino acid or the mutational pair with the ionic power source.

## Results

### Phylogeny of MotB and Node Composition of MotB-ASRs

**FIGURE 1:**
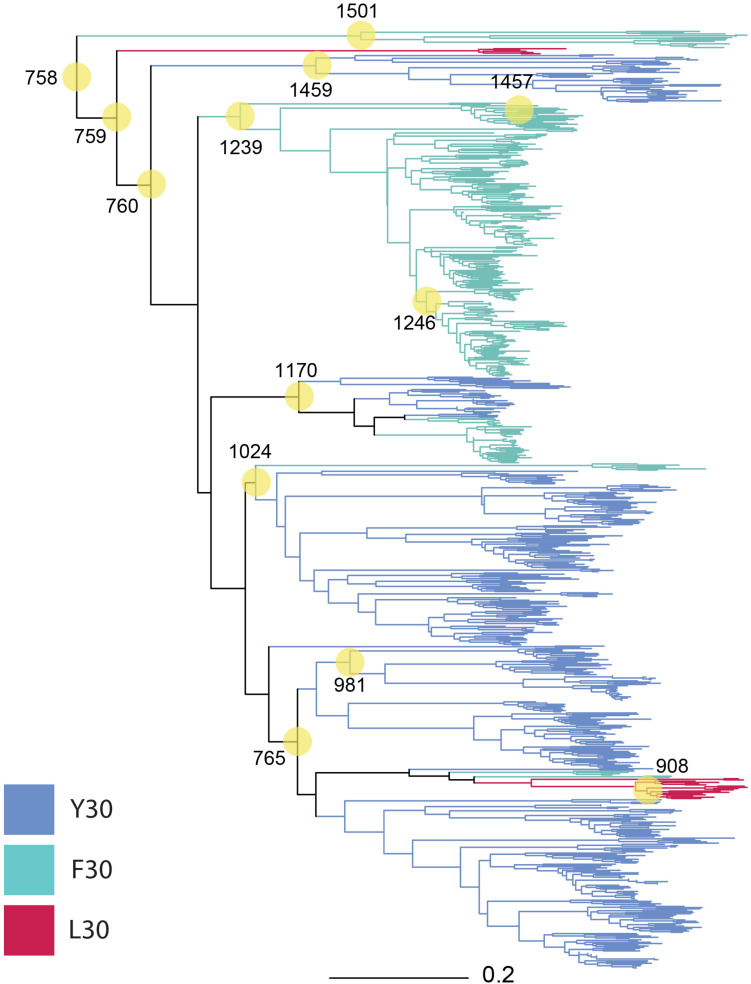
Phylogeny of 757 MotB homologs. MotB homologs selected via EBI-EST and restricted to FAD/YAD/LAD at residues 30–32 (*E. coli* MotB). ASRs were calculated for every node, with those selected for engineering and resurrection indicated (yellow circles, numbering from root beginning at 758). Tree branches were colored based on sequence identity at residue 30 F/Y/L in the contemporary strain where conserved (Y30: blue, F30: green, L30: red, mixture: black).

We selected nodes in our phylogeny (Figure 1) for ancestral reconstruction (ASR) by a mixture of early and contemporary nodes to increase the probability of synthesizing a motile gene. Among the selected nodes, ASR981 had descendants that were exclusively betaproteobacteria, ASR1459 had descendants that were exclusively alphaproteobacteria, ASR908, ASR1170, ASR1239, ASR1246, ASR1457, and ASR1501 had descendants that were exclusively gammaproteobacteria, and the remaining, mostly older nodes ASR758, ASR759, ASR760, ASR765, and ASR1024 had descendants that were a mixture of gamma, beta and alphaproteobacterial, with ASR1024 also including all four of the hydrogenophilalias.

**FIGURE 2:**
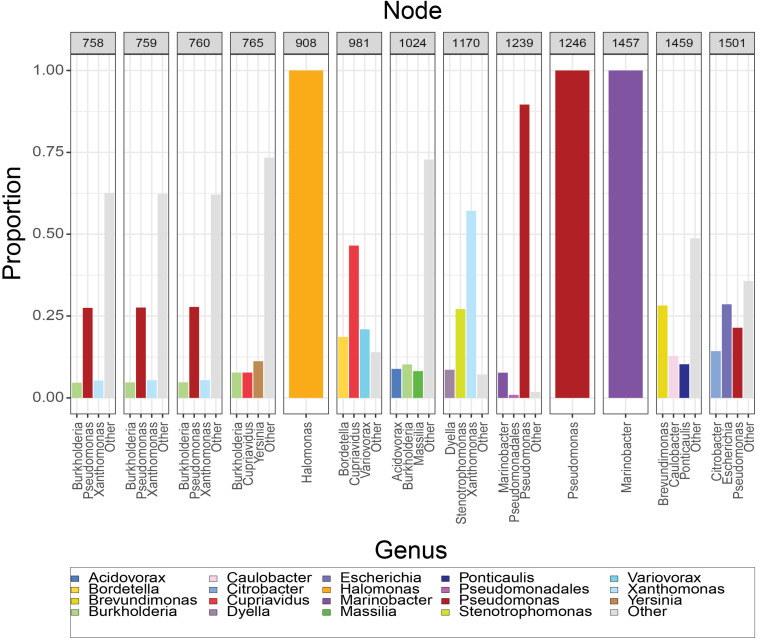
Composition of resurrected MotB-ASRs. For each node, the set of tips descendant from that node was used to calculate the proportion of genera downstream from that node. Majority genera are indicated in legend and the top three contributors to each node noted on axis. Nodes 908, 1246, and 1457 have descendants that are solely *Halomonas*, *Pseudomonas* and *Marinobacter*, respectively, while others are mixtures.

The composition of the 13 nodes is shown as the proportion of species represented in the tips downstream from that node (Figure 2). All the nodes were distributed to a wide range of bacterial genera among which *Pseudomonas, Xanthomonas, Burkholderia, Yersinia, Paraburkholderia, Cupriavidus, Stenotrophomonas, Marinobacter, Xenorhabdus*, and *Caballeronia* were prevalent. We calculated the top three genera by frequency of occurrence in the tips descendant from the selected nodes. Among all the 13 MotB-ASRs, all the descendants of ASR908 were *Halomonas*, all of ASR1246 were *Pseudomonas*, all of ASR1457 belonged to *Marinobacter*, and remaining nodes had descendants from mixed genera (Figure 2). With regard to species that could be expected to be sodium swimmers, in our original collection of 2187 MotB homologs we had 72 *Vibrio* species and 13 *Shewanella*, species. Following our network-based selection of refined sequences (Supplementary Figure 1), seven *Vibrio* strains remained in our phylogeny, and ASR1459 was the nearest parental-node to these seven strains.

### Functional Characteristics of MotB-ASR Proteins

**FIGURE 3:**
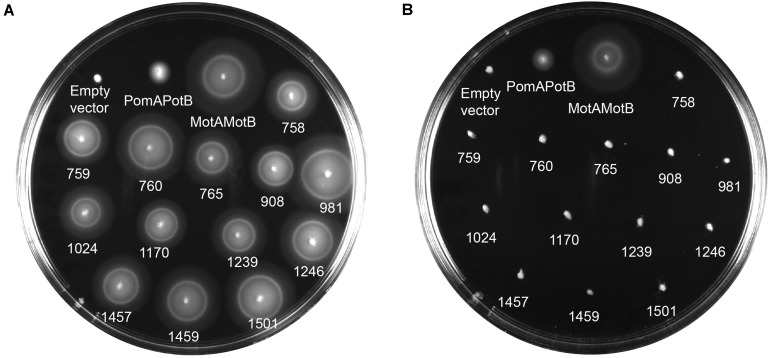
MotB-ASRs are functional. Motility of resurrected MotB-ASRs along with the control Na^+^ swimmer (PomAPotB), H^+^ swimmer (MotAMotB), and non-swimmer (empty vector: pBAD33) were tested on semi-solid agar swim plates (0.25% w/v agar). **(A)** All MotB-ASRs (presented according to the respective nodes) restore motility in the presence of PomA (from plasmid) and MotA (from genome) in Δ*motB E. coli* strain RP3087. **(B)** None of the resurrected MotB-ASRs restore motility in presence of PomA without MotA in Δ*motAmotB E. coli* strain RP6894. Plates were incubated at 30°C for 14 h.

We calculated MotB-ASRs using maximum likelihood methods (Yang, 2007) for each of the nodes above and engineered the TM and plug domains of these ASRs into our existing MotB chassis (Supplementary Figure 2). Motility, initially evaluated with swim plates, showed that all MotB-ASR proteins were functional and could restore motility in the presence of contemporary MotA (Figure 3A and Supplementary Figure 3). However, without MotA, in the presence of existing PomA, none of the MotB-ASRs were functional (Figure 3B). This is in contrast with the controls where PotB (chimeric B-unit) was functional together with PomA and non-functional with MotA (Supplementary Figure 4).

### Ion Dependency of Motile MotB-ASRs

**FIGURE 4:**
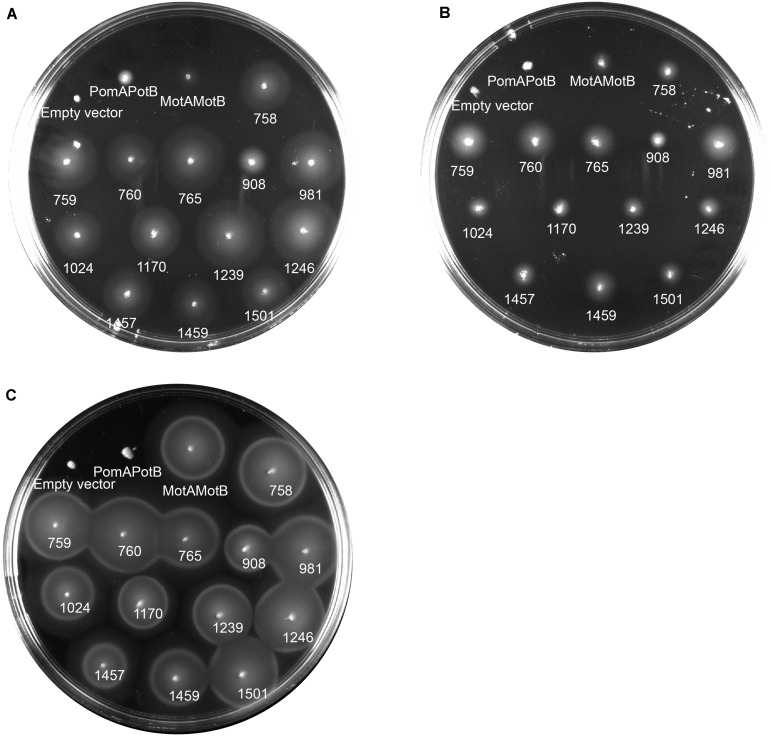
Ion dependency of MotB-ASRs. Characterizing ion dependency of motile MotB-ASRs in the presence of Na^+^ control (PomAPotB) and H^+^ control (MotAMotB) on minimal swim plates (with/without NaCl). **(A)** All MotB-ASRs (presented according to the respective nodes) restored motility in Δ*motB* strain RP3087 in the presence of Na^+^. **(B)** In the absence of Na^+^, all the MotB-ASRs restored motility in Δ*motB* strain RP3087. **(C)** All MotB-ASRs restored motility in the presence of Na^+^ and 100 μM of the sodium-blocker phenamil. **(A,B)** incubated at 30°C for 16 h, **(C)** at 30°C for 14 h.

To determine the ion dependency of motile MotB-ASRs, we measured motility both in the presence and absence of Na^+^ on minimal swim plates. The minimal swim plate assay results showed that all the ASRs were able to swim in both the presence and absence of Na^+^ (Figures 4A,B). We further checked the swimming ability of all ASRs and H^+^/Na^+^ swimming controls (MotAMotB/PomAPotB) in the presence of the Na^+^ blocker phenamil. The results showed no inhibition of motility for all MotB-ASRs and the control H^+^ swimmer, but showed complete inhibition of motility for the control Na^+^ swimmer (PomAPotB) (Figure 4C). However, the swimming diameter of all MotB-ASRs and proton and sodium controls (MotAMotB and PomAPotB), was greater at higher Na^+^ concentrations (Supplementary Figure 5). This occurred despite the observed growth rates for MotB-ASRs and controls being equivalent at all sodium concentrations (Supplementary Figure 6). To verify the ionic energy source, we characterized stator energization via tethered cell assays.

### Effect of Na^+^ on the Rotational Speed of MotB-ASRs

**FIGURE 5:**
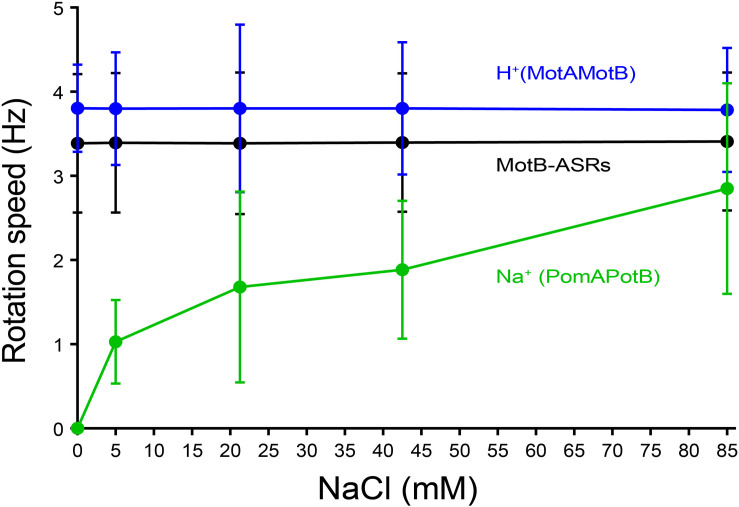
Tethered cell rotational speeds of MotB-ASRs vs. extracellular concentration of Na^+^. The averaged tethered cell rotation speed (mean ± SD) of all ASR measurements shows no dependency on external sodium concentration. Full data for each ASR is shown in Supplementary Figure 7. Rotation speed of the sodium powered swimming control (PomAPotB, green) and proton powered swimming control (MotAMotB, blue) are indicated for comparison.

The rotational speed of all 13 MotB-ASRs and the control strains (Na^+^ swimmer and H^+^ swimmer) were measured in the presence of different Na^+^ concentrations (0 mM, 5 mM, 21.25 mM, 42.5 mM, and 85 mM) using the tethered cell assay. Rotation speed of all tested MotB-ASRs showed no dependence on Na^+^ concentration similar to the H^+^ control swimmer (MotAMotB) (Figure 5). However, the Na^+^ control swimmer (PomAPotB) showed sodium dependence, with a gradual increase in swimming speed starting from 0 Hz to 2.8 ± 1.25 Hz maximum speed in the presence of 0 mM and 85 mM NaCl, respectively (Figure 5).

Among all 13 MotB-ASRs, MotB-ASR981 showed the highest swimming speed of 4.01 ± 0.92 Hz and MotB-ASR908 showed the lowest swimming speed of 2.71 ± 0.51 Hz. The remaining MotB-ASRs showed swimming speeds between 3.07 ± 0.55 Hz and 3.73 ± 0.92 Hz in all tested concentrations of Na^+^ (Supplementary Figure 7).

### Compatibility of MotB-ASRs With Ancient *Aquifex aeolicus* MotA

**FIGURE 6:**
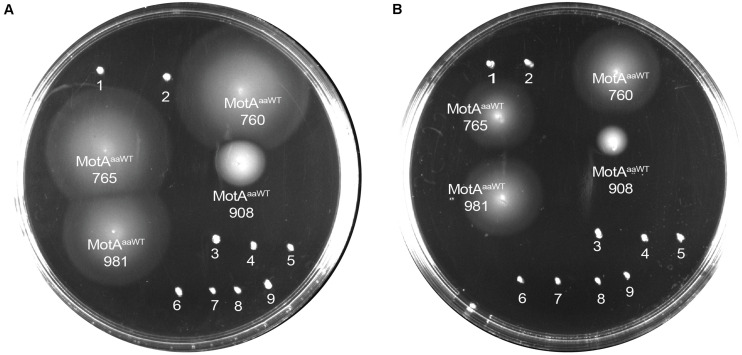
Compatibility of MotB-ASRs with *Aquifex aeolicus* MotA^*aaWT*^ on minimal swim plates (with/without NaCl). Of the 13 MotB-ASRs, four restored motility in combination with MotA^*aaWT*^ in both the presence of Na^+^ **(A)** and in the absence of Na^+^ **(B)**. Legend: 1, MotA^*aaWT*^MotB-ASR758; 2, MotA^*aaWT*^MotB-ASR759; 3, MotA^*aaWT*^MotB-ASR1024; 4, MotA^*aaWT*^MotB-ASR1170; 5, MotA^*aaWT*^MotB-ASR1239; 6, MotA^*aaWT*^MotB-ASR1246; 7, MotA^*aaWT*^MotB-ASR1457; 8, MotA^*aaWT*^MotB-ASR1459; 9, MotA^*aaWT*^MotB-ASR1501.

We co-transformed all the 13 MotB-ASRs with an ancient wild type (WT) *Aquifex aeolicus* (aa) MotA (MotA^*aaWT)*^ into a stator deleted *E. coli* (RP6894) to evaluate compatibility between *A. aeolicus* and MotB-ASR stators using the swim plate motility assay. Four (MotB-ASR760, MotB-ASR765, MotB-ASR908, and MotB-ASR981) out of 13 MotB-ASRs were compatible with MotA^*aaWT*^. All the compatible MotB-ASRs showed Na^+^ independent motility and swam both in the presence and absence of Na^+^ (presence of K^+^) containing plates (Figures 6A,B). However, the same MotA^*aaWT*^ did not show any compatibility with contemporary *E. coli* MotB (MotB^*E*^) (Supplementary Figure 8A). On the other hand, the point mutant (A225D) of *A. aeolicus* MotA (MotA^*aa*225*D*^) was compatible with *A. aeolicus-E. coli* chimeric MotB (MotB^*AE*^) and produced a much larger swimming zone than MotA^*aaWT*^ and the identical MotB^*AE*^ (Supplementary Figure 8B). In contrast, no MotB-ASRs were compatible with the point mutant of MotA^*aa*225*D*^ and all were non-motile (Supplementary Figure 8C).

### Confirmation of Na^+^ Independence of *A. aeolicus* Compatible Motile MotB-ASRs

**FIGURE 7:**
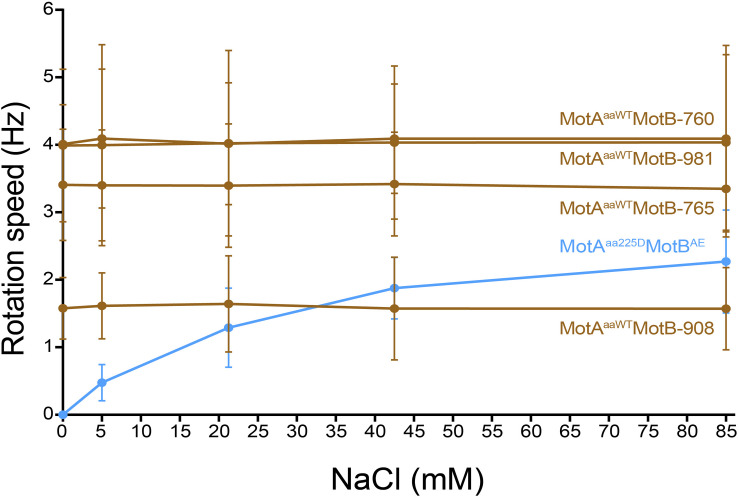
Tethered cell rotational speeds of MotA^*aaWT*^MotB-ASRs vs. extracellular concentration of Na^+^. Rotational speed measured by tethered cell for varying extracellular concentrations of Na^+^ showed that the four *A. aeolicus* compatible MotB-ASRs rotated independently of external concentration of Na^+^ (MotB-ASR908, MotB-ASR765, MotB-ASR981, MotB-ASR760, dark brown colored lines). This is in contrast to the control sodium-motile *A. aeolicus* point mutant MotA in combination with *A. aeolicus-E. coli* chimeric MotB (MotA^*aa*225*D*^ MotB^*AE*^, blue line).

Tethered cell assay with various concentration of Na^+^ (0 mM, 5 mM, 21.25 mM, 42.5 mM, and 85 mM) was performed to confirm the ionic energy source of the four motile MotB-ASRs (MotB-ASR760, MotB-ASR765, MotB-ASR908, and MotB-ASR981) together with MotA^*aaWT*^ co-transformed cells. We also measured the rotation speed of MotA^*aa*225*D*^ and MotB^*AE*^ co-transformed cell in the same condition as a Na^+^ dependent control (Takekawa et al., 2015). The rotation speeds of all the cells with MotB-ASRs and MotA^*aaWT*^ were stable and showed no dependence on external sodium concentration, while in contrast the rotation of MotA^*aa*225*D*^ and MotB^*AE*^ showed Na^+^ dependence (Figure 7). MotA^*aaWT*^MotB-ASR908 rotated more slowly than MotA^*aaWT*^MotB^*AE*^ with an average swimming speed of 1.59 ± 0.60 Hz but all other MotB-ASRs rotated more quickly, with MotB-ASR765 the fastest swimmer with the average swimming speed of 3.39 ± 0.79 Hz (Figure 7).

### Pairwise Correlation of Residue With Phenotype

We assembled a sequence alignment including all 13 MotB-ASRs (Supplementary Figure 2A) and 19 additional bacterial species (Supplementary Table 3) with known sodium or proton motility (Ishida et al., 2019). We examined if the addition of our 13 proton-motile strains affected significance scores for correlation between specific residues and phenotype by Fisher’s Exact Test. For the hypothesized correlation between residue 30 F or Y (Figure 1) and proton or sodium motility, where the null hypothesis of no correlation was previously strongly rejected based on data from 19 living strains (*p* = 0.0034), the addition of 13 additional proton motile ASR sequences weakened, but did not completely eliminate, statistical significance at the *p* < 0.05 level (*p* = 0.014). However, residue 43 remained strongly significantly correlated with ionic power source (*p* = 0.00041 with living sequences; *p* = 0.000028 with ASRs added, Supplementary Table 4).

## Discussion

The bacterial flagellar motor is a nanomachine powered by the translocation of specific ions across the cell membrane (Sowa and Berry, 2008). Initially, H^+^ and Na^+^ using bacteria were the primary interests of study but the gradual discovery of bacteria utilizing other ions, such as K^+^, Rb^+^, Mg^2+^, Ca^2+^, or Sr^2+^, has raised the question about the origin and mechanism of the ion selectivity of bacterial flagellar motors (Li et al., 2011; Terahara et al., 2012; Minamino and Imada, 2015; Imazawa et al., 2016; Ito and Takahashi, 2017). The reliable answer to this question is still not clear as it is not known whether the original swimmer was sodium or proton powered (Schirrmeister et al., 2015). The development of ancestral reconstruction has created the opportunity to recreate historical scenarios using statistical phylogenetics and microbial engineering (Hochberg and Thornton, 2017). In this study, we tried to reconstruct the ancestral MotB protein and engineered them to evaluate their characteristics.

For the reconstruction of ancestral MotB stator protein, we generated a phylogeny of 757 MotB homologs. We focused on engineering the TM and plug region of ancestral MotB as this region controls ion selectivity and has been engineered to make functional chimeras (Asai et al., 2000; Ito et al., 2005; Nishino et al., 2015). Our phylogenies were restricted to include essential D32 to ensure functionality, and we further restricted the phylogenies to include FAD/YAD/LAD in residues 30–32 to have the best chance of generating functional reconstructions, and to examine the effect of residue changes and adaptation at 30F/Y/L to probe correlation with H^+^ or Na^+^ ion selection and compare with previous work (Ishida et al., 2019; Supplementary Figure 2B).

The swim plate assay results of this study showed that all the MotB-ASRs were able to swim both in the presence and absence of Na^+^ in the combination with MotA. Also, our MotB-ASRs were not inhibited by the sodium inhibitor phenamil that is a general inhibitor of sodium dependent motors (Atsumi et al., 1990), and amiloride and its derivatives do not inhibit MotAB (Terahara et al., 2008). Na^+^ independent motility and resistance to Na^+^ blocker together imply that our ancestral B-subunits function similarly to proton-powered MotB, and our alterations in the TM domain converted a Na^+^ dependent swimmer (PomAPotB) into a sodium independent swimmer (MotAPomAMotB-ASR).

Tethered cell assay results confirmed that external Na^+^ concentrations did not affect the rotation speed of all MotB-ASRs (Figure 5), whereas the rotation speed of the control Na^+^ swimmer showed a strong dependence on external sodium concentration (Figure 5). The sodium-independence of MotB-ASR swimming speed matches previous measurements for proton-powered MotAB (Imazawa et al., 2016). This result indicates that our resurrected reconstructions are more MotB-like than PomB-like.

Our MotB-ASRs included both YAD and FAD motifs, however, regardless of F/Y at residue 30, all MotB-ASRs displayed proton-based functionality. Previously we quantified the correlation between F/Y and sodium/proton motility in a test set of known sodium and proton swimmers (Ishida et al., 2019). There are known exceptions to the overall trend, such as proton-motile *Pseudomonas aeruginosa* with a FAD motif, but the overall correlation was strong, and both generalized and phylogenetic linear regressions indicated significant correlation (*p* < 0.05) in our previous work. We reexamined our previous data set, and supplemented this with our new ASRs. When we added the new sequences corresponding to our ASRs, which were diverse in F/Y but uniform in phenotype, this resulted in the F/Y locus, in our analysis, having a much weaker correlation with a sodium/proton phenotype. This is further evidence that FAD/YAD in MotB is unlikely to dictate selectivity on its own, alongside ours and others’ previous work (Asai et al., 2000, 2003; Sudo et al., 2009).

Additionally, the compatibility of our MotB-ASR with ancient *Aquifex aeolicus* MotA (MotA^*aaWT*^) and subsequent Na^+^ independent swimming properties provided further evidence that the MotB-ASRs generated here are more likely to be proton-powered MotB. In agreement, our ASRs could not restore motility with the mutant *A. aeolicus* MotA (MotA^*aa*225*D*^) which had been previously shown to enable sodium-dependent motility (Takekawa et al., 2015).

The primacy of sodium energetics is becoming clear in the case of ATPases (Mulkidjanian et al., 2008), but for flagellar motors this is still an open question. Here, we observed our ASRs to function in a MotB-like sodium-independent manner, and to be functional in the presence of (MotA^*aaWT)*^. Previous work has suggested sodium-energized flagellar rotation predates proton-powered bacterial motion, due to the early diverging *A. aeolicus* being observed to be sodium-dependent (Takekawa et al., 2015). We have not attempted here the difficult task of definitively resolving ancient evolutionary history. To answer which ion holds energetic primacy for the BFM would require more comprehensive phylogenies, across multiple flagellar proteins, to identify and resurrect the best estimate of the historical stator complex at the time of divergence. Here we have demonstrated that such approaches show strong promise: ancestral reconstruction and protein engineering can generate novel stators that are both functional and useful and can be used to probe questions surrounding which residues dictate ion-selectivity.

## Data Availability Statement

The datasets presented in this study can be found in online repositories. The names of the repository/repositories and accession number(s) can be found in the article/Supplementary Material.

## Author Contributions

MI and MB designed the experiments and executed molecular biology and microbiology. AL and MB executed bioinformatics surrounding SSNs, correlation, and phylogenetics. NM executed bioinformatics surrounding phylogenetics. MB supervised the design, execution and writing of the project. All authors contributed to writing and revision of the manuscript.

## Conflict of Interest

The authors declare that the research was conducted in the absence of any commercial or financial relationships that could be construed as a potential conflict of interest.

## Supplementary Material

Supplementary Figure 1 Sequence similarity network (SSN) of MotB homologs. Visualization of the SSN for the 757 sequences that composed the phylogeny shown in Figure 1. Edges indicate sequence identity higher than 85%, clustering visualized using the gamma-organic layout on Cytoscape 3.1. Class is indicated by shape (gammaproteobacteria: triangle; alphaproteobacteria: diamond; betaproteobacteria: circle; hydrogenophilalia: square) with residue identity at site 30 indicated in color as per Figure 1 (Y30: blue; F30: green; L30: red).

Supplementary Figure 2 Sequence alignment and conservation of ASRs. **(A)** Sequence alignment of 13 resurrected MotB-ASRs and selection of MotB/PomB from 19 species as a representative selection of sodium/proton motile stators, for comparison. **(B)** Sequence logo for MotB-ASRs. The height of each letter is proportional to its frequency, and the height of the entire stack signifies the information content of the sequences at that position (in bits).

Supplementary Figure 3 Swimming in semi-solid agar of MotB-ASRs. Bars represent mean ± SD of swimming diameter for 13 MotB-ASRs and three controls (empty vector: pBAD33, Na^+^: PomAPotB, H^+^: MotAMotB). The diameters were measured from the respective 0.25% LB agar + 85 mM NaCl swim plates, incubated at 30°C for 14 h (representative shown in Figure 3). Experiments performed in triplicate for the controls in duplicate for MotB-ASRs.

Supplementary Figure 4 Swimming capabilities of different MotB-ASRs. Chimeric B-subunit, PotB was functional with Na^+^ powered A-subunit, PomA but was non-functional with H^+^ powered Aa-subunit MotA. However, MotB-ASRs were functional with H^+^ powered Aa-subunit MotA.

Supplementary Figure 5 Swimming diameter vs. external Na^+^ concentration. Bars represent swimming diameter of MotB-ASRs and swimming controls (Na^+^ swimmer/H^+^ swimmer) with the change of NaCl concentration (0 mM NaCl: brown; 21.25 mM NaCl: blue; 85 mM NaCl: gray). Experiments performed in triplicate for the controls in duplicate for MotB-ASRs (additional plates and measurements for 85 mM NaCl data, not replicates of Supplementary Figure 3).

Supplementary Figure 6 Growth curves for MotB-ASRs and controls vs. external NaCl concentration. Growth curves of ASRs and controls were measured in the presence of three different NaCl concentration (0 mM, 21.25 mM, and 85 mM).

Supplementary Figure 7 Rotation of MotB-ASRs is sodium-independent. Rotational speeds of MotB-ASRs were measured using the tethered cell assay at varying concentrations of NaCl (0 mM, 5 mM, 21.25 mM, 42.50 mM, 85 mM). Colored lines represent the mean ± SD rotation speed of each MotB-ASR (*N* = 20 cells). MotB-ASR908 rotated at the lowest mean speed (sky blue) and MotB-ASR981 rotated at the greatest mean speed (green line).

Supplementary Figure 8 Compatibility of different stator units from *Aquifex aeolicus* and MotB-ASRs. **(A)** MotA^*aaWT*^ MotB^*E*^ does not swim on sodium plate. **(B)** MotA^*aaWT*^ MotB^*AE*^ does not swim while and MotA^*aa225D*^MotB^*AE*^ does swim on sodium plate. **(C)** MotA^*aa225D*^MotB-ASRs all do not swim on sodium plate. All plates are LB agar with 85 mM NaCl, 0.02% arabinose, **(A,B)** with kanamycin/ampicillin, **(C)** with chloramphenicol, as per methods.
