# Corrigendum: Ancestral Sequence Reconstructions of MotB Are Proton-Motile and Require MotA for Motility

**DOI:** 10.3389/fmicb.2021.650373

**Published:** 2021-03-19

**Authors:** Md Imtiazul Islam, Angela Lin, Yu-Wen Lai, Nicholas J. Matzke, Matthew A. B. Baker

**Affiliations:** ^1^School of Biotechnology and Biomolecular Sciences (BABS), University of New South Wales, Sydney, NSW, Australia; ^2^School of Biological Sciences, University of Auckland, Auckland, New Zealand; ^3^CSIRO Synthetic Biology Future Science Platform, Brisbane, QLD, Australia

**Keywords:** motility, flagellar and chemotaxis, stator, ancestral sequence reconstruction, ion-selectivity

In the original article, in the **Introduction**, **paragraph four**, we referenced previous work on ancestral reconstruction. We would like to add a further sentence and three additional citations for the work that first demonstrated the resurrection of ancient genes into contemporary hosts to form ancient modern hybrids and examine function *in vivo*:

It is possible to then resurrect the ancestral proteins corresponding to these inferred sequences and characterize their *in vitro* biological and biochemical properties (Gaucher et al., 2003; Thornton et al., 2003). Furthermore, ancient proteins can be expressed in contemporary hosts to examine the function of ancient proteins *in vivo*, and their integration and subsequent adaptation in ancient-modern hybrids (Kaçar and Gaucher, 2012; Kacar et al., 2017a,b).

Whilst our work does not explore subsequent adaptation of these ancient-modern hybrids, citation of these references is appropriate to give appropriate credit and to guide the reader to consider the research in ancestral reconstruction that has enabled our work.

The authors apologize for this error and state that this does not change the scientific conclusions of the article in any way. The original article has been updated.
