# Supplementary material for: Ancestral Sequence Reconstructions of MotB Are Proton-Motile and Require MotA for Motility
Source: Front Microbiol. 2020 Dec 23;11:625837. doi: 10.3389/fmicb.2020.625837 (PMC7787011; doi:10.3389/fmicb.2020.625837)
Supplement: Supplementary Figure 1 — Sequence similarity network (SSN) of MotB homologs. Visualization of the SSN for the 757 sequences that composed the phylogeny shown in Figure 1. Edges indicate sequence identity higher than 85%, clustering visualized using the gamma-organic layout on Cytoscape 3.1. Class is indicated by shape (gammaproteobacteria: triangle; alphaproteobacteria: diamond; betaproteobacteria: circle; hydrogenophilalia: square) with residue identity at site 30 indicated in color as per Figure 1 (Y30: blue; F30: green; L30: red). [file Data_Sheet_1.pdf]

Supplementary Information

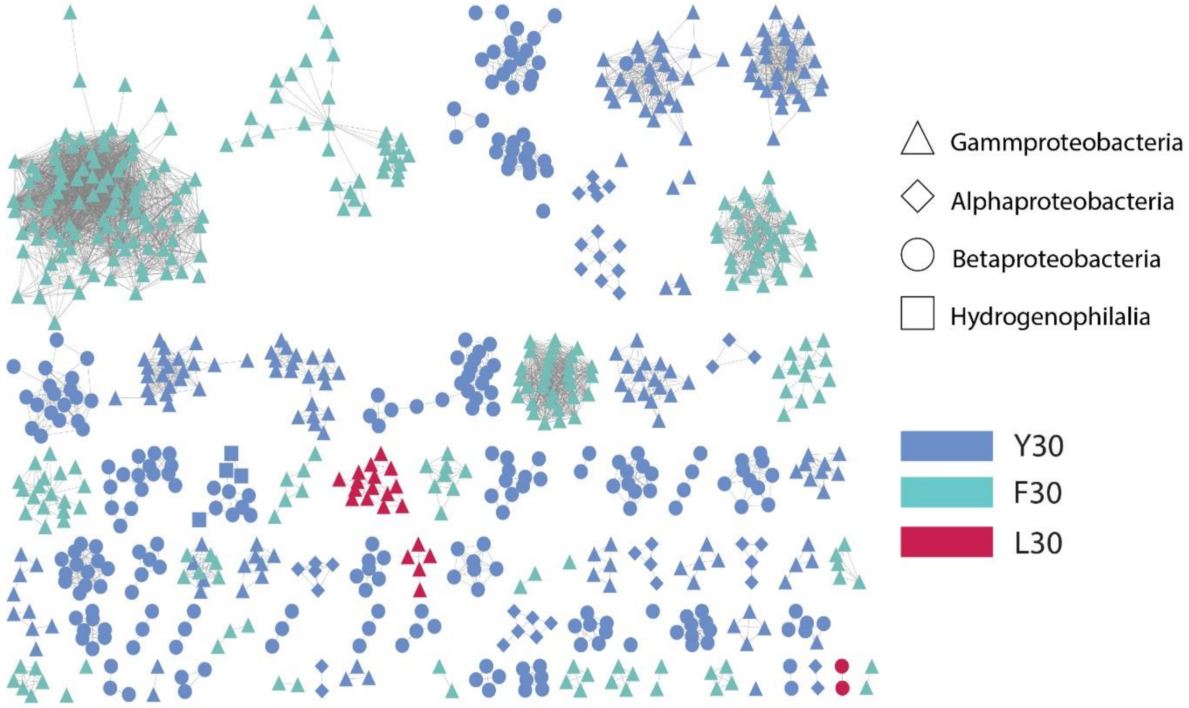

Supplementary Fig. 1. Sequence similarity network (SSN) of MotB homologues.

Supplementary Fig. 1. Sequence similarity network (SSN) of MotB homologues. Visualisation of the SSN for the 757 sequences that composed the phylogeny shown in Fig. 1. Edges indicate sequence identity higher than 85%, clustering visualized using the gamma-organic layout on Cytoscape 3.1. Class is indicated by shape (gammaproteobacterial: triangle; alphaproteobacterial: diamond; betaproteobacteria: circle; hydrogenophilalia: square) with residue identity at site 30 indicated in colour as per Fig. 1 (Y30: blue; F30: green; L30: red).

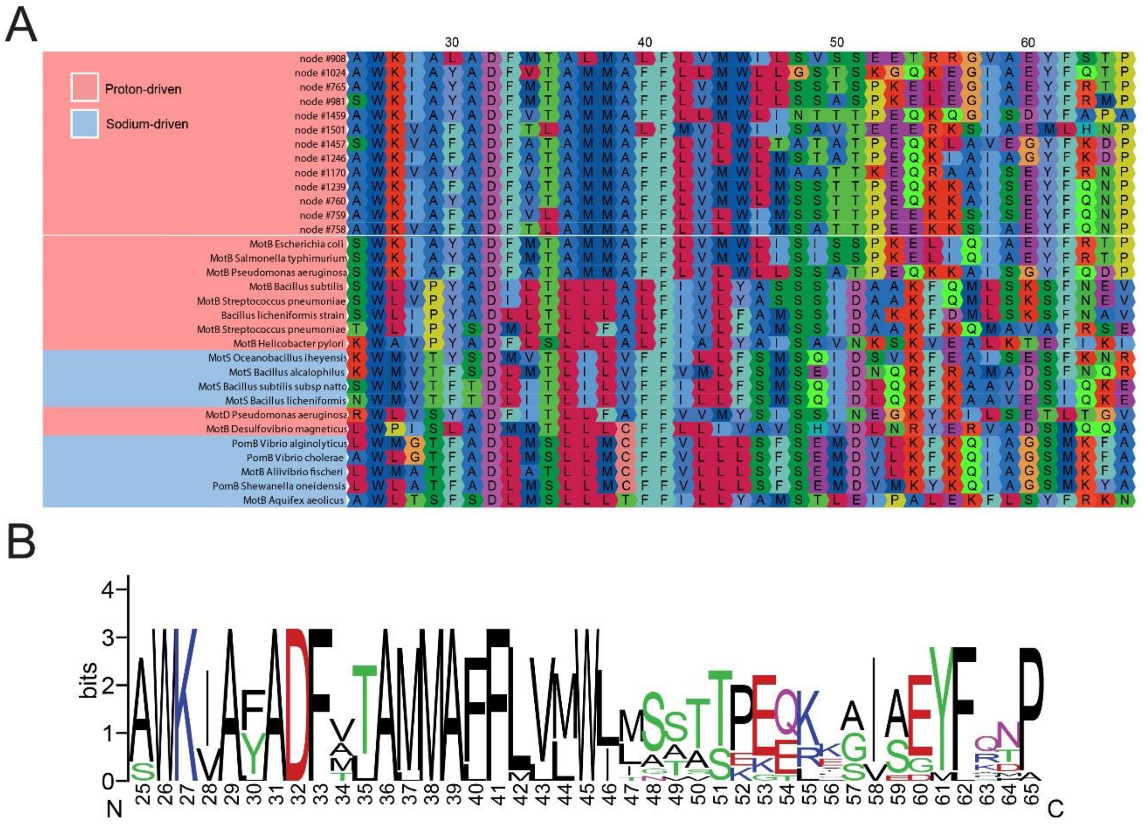

Supplementary Fig. 2. Sequence alignment and conservation of ASRs. (A) Sequence alignment of 13 resurrected MotB-ASRs and selection of MotB/PomB from 19 species as a representative selection of sodium/proton motile stators, for comparison. B) Sequence logo for MotB-ASRs. The height of each letter is proportional to its frequency, and the height of the entire stack signifies the information content of the sequences at that position (in bits).

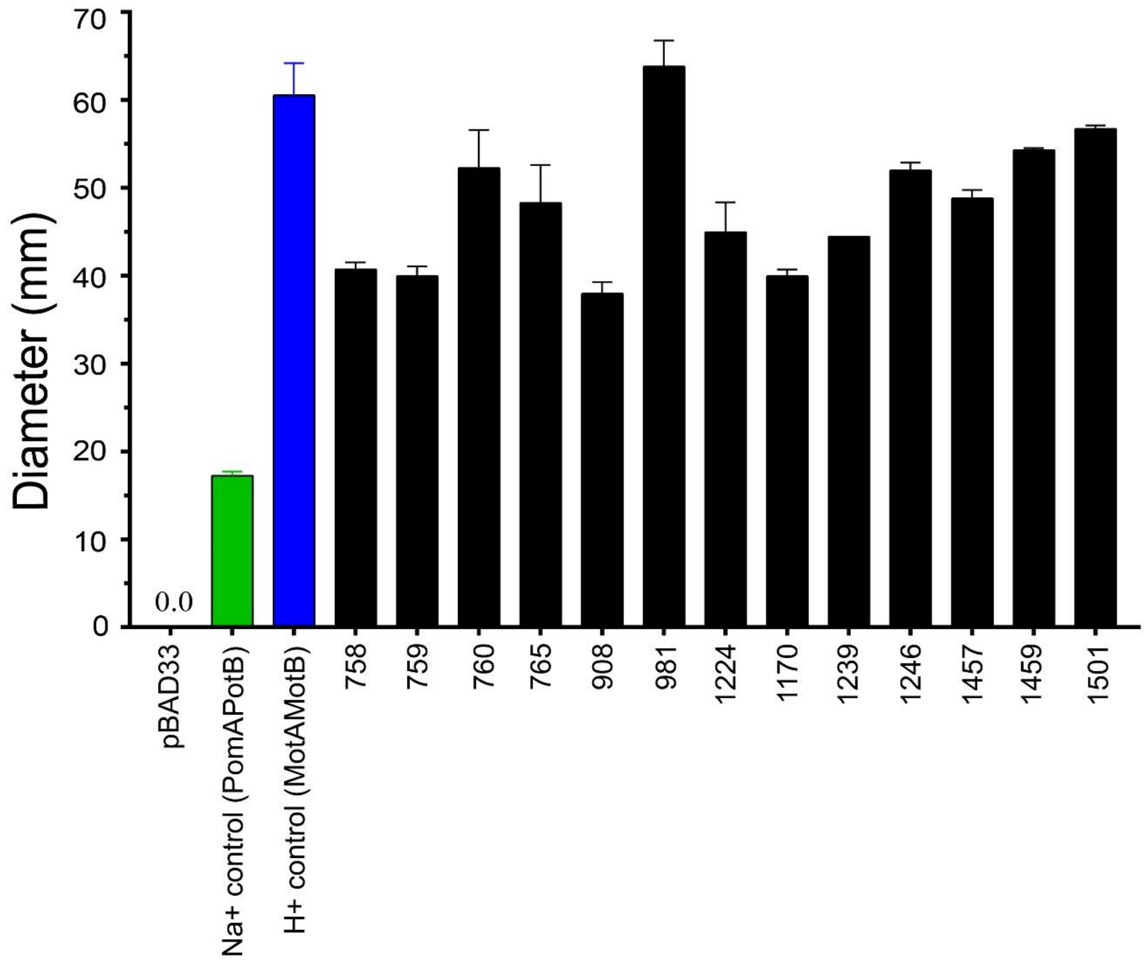

Supplementary Fig. 3. Swimming in semi-solid agar of MotB-ASRs. Bars represent mean ± SD of swimming diameter for 13 MotB-ASRs and three controls (empty vector: pBAD33, Na^+: PomAPotB, H^+: MotAMotB). The diameters were measured from the respective 0.25% LB agar + 85 mM NaCl swim plates, incubated at 30˚C for 14 hours (representative shown in Fig. 3). Experiments performed in triplicate for the controls in duplicate for MotB-ASRs.

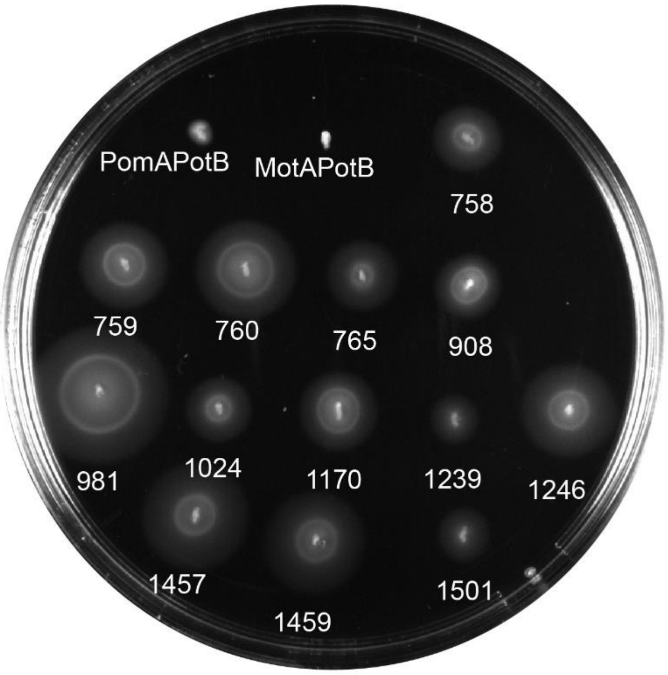

Supplementary Fig. 4. Swimming capabilities of different MotB-ASRs. Chimeric B-subunit, PotB was functional with Na^+ powered A-subunit, PomA but was non-functional with H^+ powered Aa-sub-unit MotA. However, MotB-ASRs were functional with with H^+ powered Aa-sub-unit MotA.

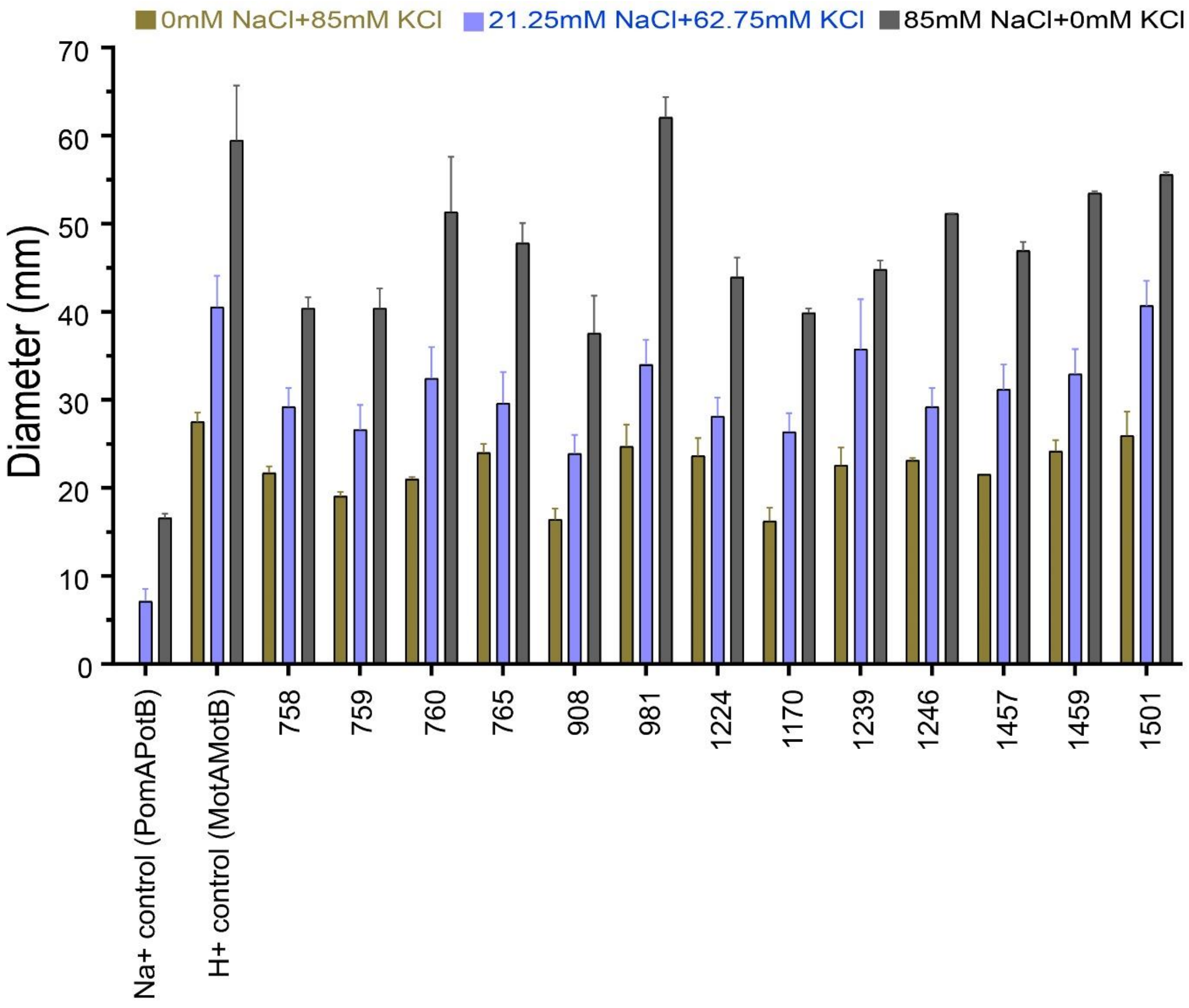

Supplementary Fig. 5. Swimming diameter vs external Na^+ concentration. Bars represent swimming diameter of MotB-ASRs and swimming controls (Na^+ swimmer/H^+ swimmer) with the change of NaCl concentration (0 mM NaCl: brown; 21.25 mM NaCl: blue; 85 mM NaCl: grey). Experiments performed in triplicate for the controls in duplicate for MotB-ASRs (additional plates and measurements for 85 mM NaCl data, not replicates of Supplementary Fig. 3).

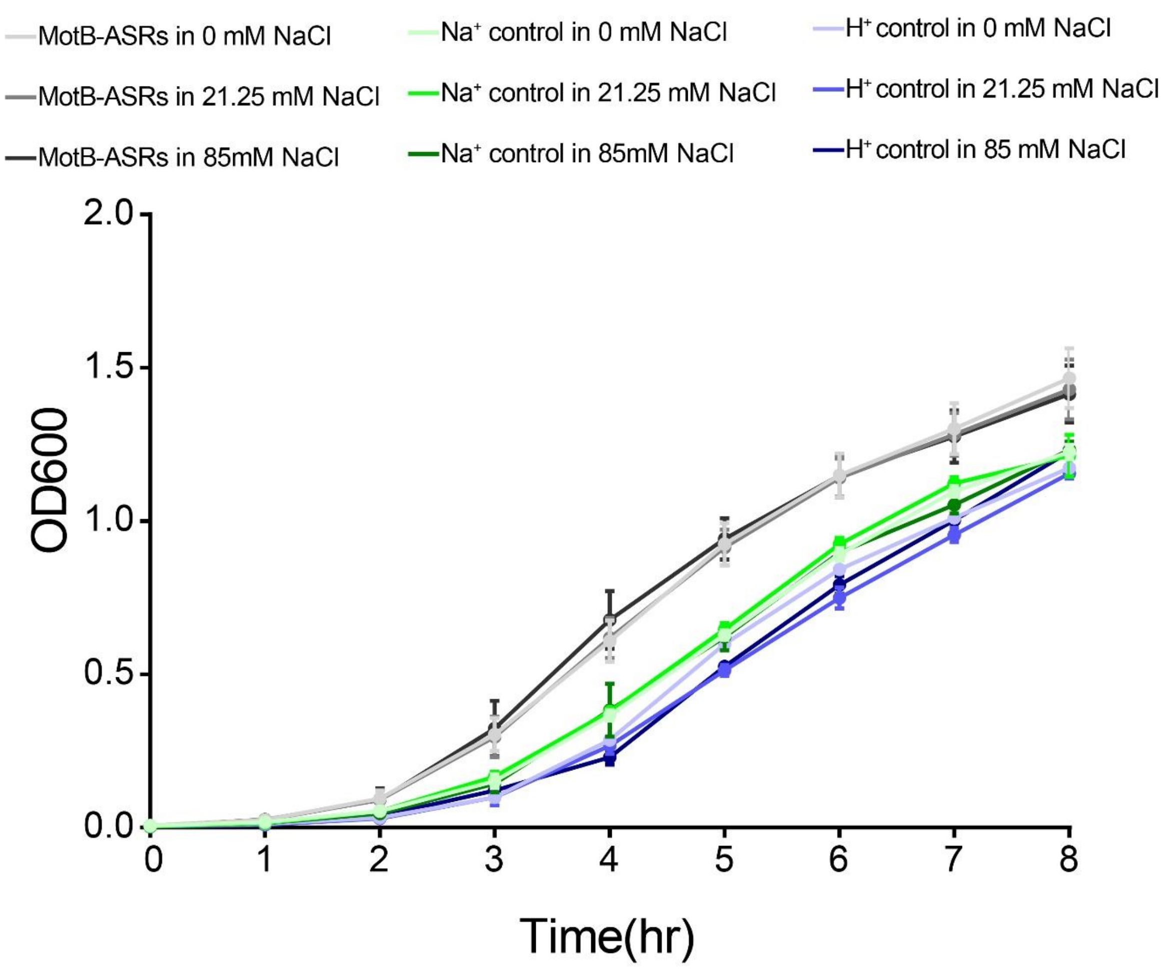

Supplementary Fig. 6. Growth curves for MotB-ASRs and controls vs external NaCl concentration. Growth curves of all MotB-ASRs and controls were measured in the presence of three different NaCl concentration (0 mM, 21.25 mM and 85mM).

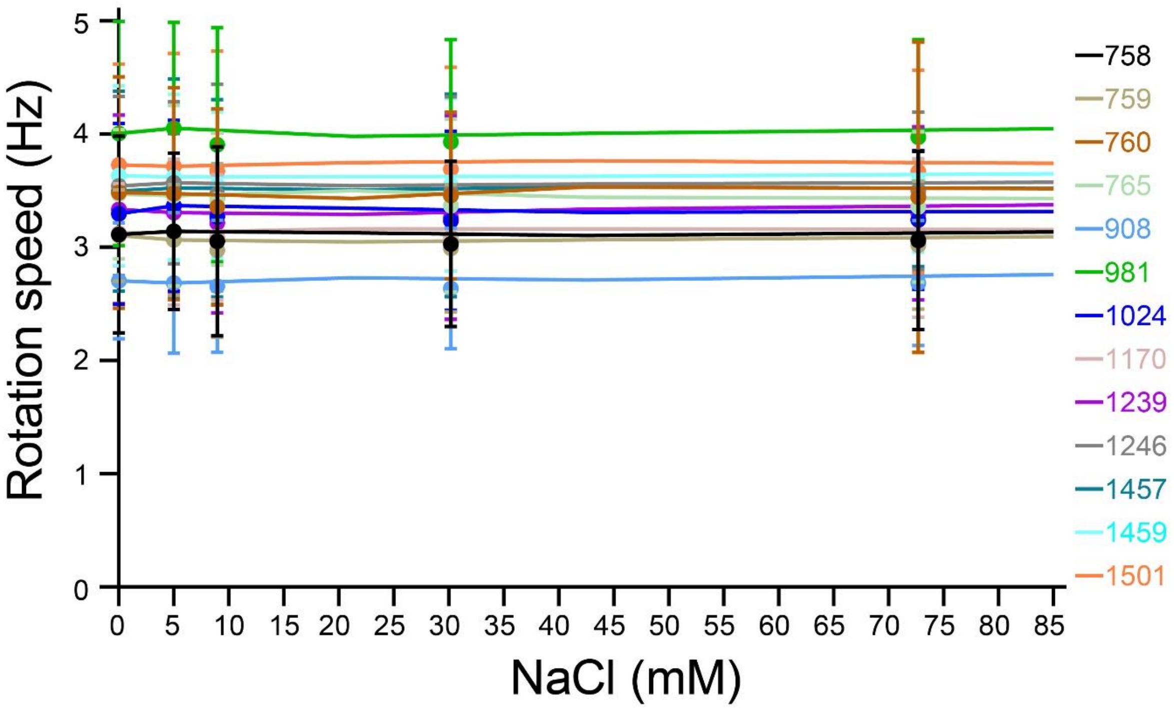

Supplementary Fig. 7. Rotation of MotB-ASRs is sodium-independent. Rotational speeds of MotB-ASRs were measured using the tethered cell assay at varying concentrations of NaCl (0 mM, 5 mM, 21.25 mM, 42.50 mM, 85 mM). Coloured lines represent the mean ± SD rotation speed of each MotB-ASR (N = 20 cells). MotB-ASR908 rotated at the lowest mean speed (sky blue) and MotB-ASR981 rotated at the greatest mean speed (green line).

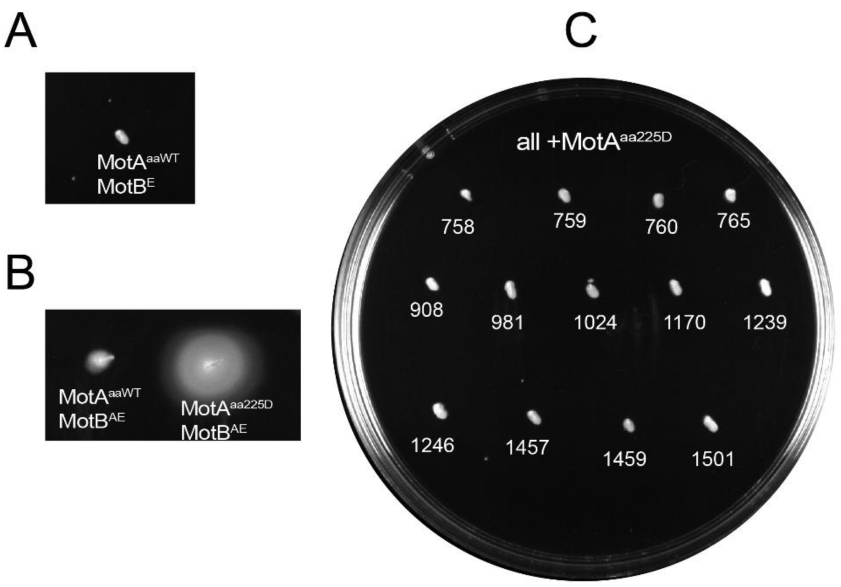

Supplementary Fig. 8. Compatibility of different stator units from *Aquifex aeolicus* and MotB-ASRs. **(A)** MotA^{aaWT} MotB^{E} does not swim on sodium plate. **(B)** MotA^{aaWT} MotB^{AE} does not swim while and MotA^{aa225D}MotB^{AE} does swim on sodium plate. **(C)** MotA^{aa225D}MotB-ASRs all do not swim on sodium plate. All plates are LB agar with 85 mM NaCl, 0.02% arabinose, A/B with kanamycin/ ampicillin, (C) with chloramphenicol, as per methods.

Supplementary Table 1: List of strains and plasmids used

ARA, Arabinose; CAM, Chloramphenicol, AMP, Ampicillin; KAN, Kanamycin

**Strains**	**Description**	**Reference**
RP6894	*E. coli* (ΔMotA, ΔMotB)	J. S. Parkinson (Block et al., 1989)
RP3087	*E. coli* (ΔMotB)	J. S Parkinson (Blair et al., 1991)
**Plasmids**	**Description**	**Reference**
pSHU1234	PomA and PotB, Ara, CAM^R	(Kojima et al., 2008)
pBAD33	Empty vector, CAM^R	(Guzman et al., 1995)
pDB108	MotA and MotB, CAM^R	David F Blair
pMotB	pDB108 ΔMotA CAM^R	This study
pPotB	pSHU1234 ΔPotB CAM^R	This study
pPomA	pSHU1234 ΔPomA CAM^R	This study
pNT7	Wild type *A. aeolicus* MotA, AMP^R	(Takekawa et al., 2015)
$pMotA^{aa225D}$	A225D Point mutant *A. aeolicus* MotA, AMP^R	This study and (Takekawa et al., 2015)
pNT11	pSBETa- $motB_2^{AE}$, KAN^R	(Takekawa et al., 2015)
p758	PomA and MotB-ASR758, pSHU1234 backbone, CAM^R	This study
p759	PomA and MotB-ASR759, pSHU1234 backbone, CAM^R	This study
p760	PomA and MotB-ASR60, pSHU1234 backbone, CAM^R	This study
p765	PomA and MotB-ASR765, pSHU1234 backbone, CAM^R	This study
p908	PomA and MotB-ASR908, pSHU1234 backbone, CAM^R	This study
p981	PomA and MotB-ASR981, pSHU1234 backbone, CAM^R	This study
p1024	PomA and MotB-ASR1024, pSHU1234 backbone, CAM^R	This study
p1170	PomA and MotB-ASR1170, pSHU1234 backbone, CAM^R	This study
p1239	PomA and MotB-ASR1239, pSHU1234 backbone, CAM^R	This study
p1246	PomA and MotB-ASR1246, pSHU1234 backbone, CAM^R	This study
p1457	PomA and MotB-ASR1457, pSHU1234 backbone, CAM^R	This study
p1459	PomA and MotB-ASR1459, pSHU1234 backbone, CAM^R	This study
p1501	PomA and MotB-ASR1501, pSHU1234 backbone, CAM^R	This study

Supplementary Table 2: List of primers

**Primer Category**	**Name of Primer**	**Primer Type**	**Primer Sequence (5' to 3')**
Primers for ASR cloning	ASR 758	F	TACATGGCATATGAAGAATCAAGCGCATCCGATTATTGTCGTC AAACGACGCAAAGCCAAAAGCCACGGGGCAGCAGGCGGGGCC TGGAAAGTCGCGTTTGCCGATTTTACGTTAGCAATGATGGCGT TCTTCCTGGTCCTTTGGATCATGTCAGCCACGACCCCGGAAGA GAAGAAGTCGATTGCTGAGTATTTCCAAAACCCACTGGCGACC GCGGTGACC
	ASR 759	F	TACATGGCATATGAAGAATCAAGCGCATCCGATTATTGTCGTC AAACGACGCAAAGCCAAAAGCCACGGGGCAGCAGGCGGAGCT TGGAAGATCGCTTTCGCGGATTTCGTACTGGCGATGATGGCCT TCTTTCTGGTATTGTGGATTATGTCAAGTACGACACCAGAGGA GAAAAAATCAATCAGTGAGTATTTCCAGAATCCTCTGGCGACC GCGGTGACC
	ASR 760	F	TACATGGCATATGAAGAATCAAGCGCATCCGATTATTGTCGTC AAACGACGCAAAGCCAAAAGCCACGGGGCAGCAGGAGGCGCG TGGAAAATTGCGTATGCTGATTTCGTTACGGCGATGATGGCTTT CTTCTTGGTGATGTGGCTGATGTCGTCAACAACCCCGGAGCAG AAAAAAGCTATCTCCGAATACTTCCAGAACCCGCTGGCGACCG CGGTGACC
	ASR 765	F	TACATGGCATATGAAGAATCAAGCGCATCCGATTATTGTCGTC AAACGACGCAAAGCCAAAAGCCACGGGGCAGCAGGAGGTGCG TGGAAGATTGCATACGCGGATTTTATGACAGCTATGATGGCCT TCTTTTTGGTAATGTGGCTTTTATCAAGTACCAGCCCGAAGGAA TTGGAAGGTATTGCCGAGTATTTCCGCACTCCCCTGGCGACCG CGGTGACC
	ASR 908	F	TACATGGCATATGAAGAATCAAGCGCATCCGATTATTGTCGTC AAACGACGCAAAGCCAAAAGCCACGGGGCAGCAGGAGGTGCA TGGAAAATCGCCTTAGCAGACTTTATGACTGCGCTTATGGCGT TGTTTCTGGTAATGTGGATTTTGAGCGTATCCTCTGAAGAGACA CGTCGCGGCGTCGCTGAGTATTTCAGTACACCACTGGCGACCG CGGTGACC
	ASR 981	F	TACATGGCATATGAAGAATCAAGCGCATCCGATTATTGTCGTC AAACGACGCAAAGCCAAAAGCCACGGGGCAGCAGGTGGGAGC TGGAAAATTGCTTACGCCGATTTTATGACTGCGATGATGGCAT TCTTCTTGGTCATGTGGCTGTTAAGCAGTGCTTCGCCCAAAGAG TTAGAGGGCATTGCGGAGTATTTTCGTATGCCTCTGGCGACCG CGGTGACC
	ASR 1024	F	TACATGGCATATGAAGAATCAAGCGCATCCGATTATTGTCGTC AAACGACGCAAAGCCAAAAGCCACGGGGCAGCAGGGGGTGCC TGGAAGATCGCTTATGCAGACTTCGTAACCGCAATGATGGCCT TCTTTCTGTTAATGTGGTTGTTGGGAAGCACATCCAAGGGGCA GAAGGAGGGCATTGCTGAATACTTCCAGACTCCGCTGGCGACC GCGGTGACC
	ASR 1170	F	TACATGGCATATGAAGAATCAAGCGCATCCGATTATTGTCGTC AAACGACGCAAAGCCAAAAGCCACGGGGCAGCAGGTGGCGCA TGGAAAGTAGCTTATGCTGATTTTGTAACCGCTATGATGGCGTT TTTCCTGGTAATGTGGTTGATGGCAGCTACTACCAAGGAGCAG CGTGCGGCTATCAGCGAGTATTTCCGCAATCCCCTGGCGACCG CGGTGACC

	ASR 1239	F	TACATGGCATATGAAGAATCAAGCGCATCCGATTATTGTCGTCAAACGACGCAAAGCCAAAAGCCACGGGGCAGCAGGGGGGGCCTGGAAAATTGCCTTCGCCGATTTCGCTACCGCGATGATGGCGTTCTTCTTGGTAATGTGGCTTATGTCATCAACTACGCCAGAGCAAAAGAAGGCGATTAGCGAATACTTCCAAAATCCACTGGCGACCGCGGTGACC
	ASR 1246	F	TACATGGCATATGAAGAATCAAGCGCATCCGATTATTGTCGTCAAACGACGCAAAGCCAAAAGCCACGGGGCAGCAGGAGGTGCGTGGAAAATCGCCTTTGCGGACTTCGCTACGGCGATGATGGCCTTCTTCCTTGTGCTTTGGTTGATGTCAACGGCCACACCAGAGCAGAAGATTGCTATTGCGGGTTACTTCAAAGATCCGCTGGCGACCGCGGTGACC
	ASR 1457	F	TACATGGCATATGAAGAATCAAGCGCATCCGATTATTGTCGTCAAACGACGCAAAGCCAAAAGCCACGGGGCAGCAGGGGGTTCTTGGAAGGTAGCATTCGCGGACTTTGCAACCGCGATGATGGCGTTTTTCTTGGTACTTTGGCTTACTGCCACGGCTACCCCCGAGCAAAAGTTAGCCGTCGAAGGTTACTTCAAAGACCCCCTGGCGACCGCGGTGACC
	ASR 1459	F	TACATGGCATATGAAGAATCAAGCGCATCCGATTATTGTCGTCAAACGACGCAAAGCCAAAAGCCACGGGGCAGCAGGGGGCGCGTGGAAGATTGCCTACGCGGATTTCGTTACAGCCATGATGGCATTTTTTCTTCTTATGTGGTTAATCAATACGACAACACCAGAACAAAAGCAAGGAATCTCAGATTACTTCGCACCGGCTCTGGCGACCGCGGTGACC
	ASR 1501	F	TACATGGCATATGAAGAATCAAGCGCATCCGATTATTGTCGTCAAACGACGCAAAGCCAAAAGCCACGGGGCAGCAGGCGGCGCTTGGAAAGTAGCCTTCGCTGACTTTACGTTGGCTATGATGGCTCTTTTTATGGTCCTGTGGATCATTTCTGCTGTAACTGAGGAGGAGCGTAAGAGTATCGCTGAAATGTTGCACAATCCGCTGGCGACCGCGGTGACC
	ASR Reverse Primer	R	CATCCGCCAAAACAGCCAAGCT
Primer for colony PCR screening of ASRs	ASR MotB NT Specific	F	ATCAAGCGCATCCGATTATTGTCG
Primer for sanger sequencing confirmation of ASRs	pSHU HindIII	F	ATTGCGGATAAGCTTTCTCTTCGC
	MotB End primer	F	GATCTCGACCAGTTGATAGAGTCC

Primers for cloning out of PomA from pSHU1234	PomA InDel Pshu	F	GATGAAGATAACAAATGCGATTGTCCGCCA
	PomA InDel Pshu OH	F	CTTGGAGAATTCATATGGATGATGAAGATAACAAATGCGATTGTCCGCCA
	PomA InDel Pshu	R	AAAGCACTCCTCACGCTGTCGA
	PomA InDel OH Pshu	R	ATCCATATGAATTCTCCAAGAAAGCACTCCTCACGCTGTCGA
Primers for cloning out of PotB from pSHU1234	PotB InDel Pshu F	F	CTGTTTTGGCGGATGAGAGAAGATTTT
	PotB InDel Pshu OH F	F	CTGCAGGCATGCAAGCTTGGCTGTTTTGGCGGATGAGAGAAGATTTT
	PotB InDel Pshu R	R	ATGAATTCTCCAAGTTACTCGTCAATCTCAAGGGC
	PotB InDel OH Pshu R	R	CCAAGCTTGCATGCCTGCAGATGAATTCTCCAAGTTACTCGTCAATCTCAAGGGC
Primers for cloning out of MotA from pDB108	MotA inv-del-p108	F	CATATGGTACTCCTTATGGCATTATTGATGA
	MotA inv-del-OH-p108	F	ATGCGCTTGATTCTTCATCATATGGTACTCCTTATGGCATTATTGATGA
	MotA inv-del-p108	R	CCGATTATTGTCGTCAAACG
	MotA inv-del-OH-p108	R	ATGAAGAATCAAGCGCATCCGATTATTGTCGTCAAACG
Primers for A225D point mutation of *A. aeolicus* MotA	Aquifex MotA A225D	F	CTCCCTTCTGTATCTTTTCAATATCCTCTATGTAAATGGTCTTTACG
	Aquifex MotA A225D	R	CGTAAAGACCATTTACATAGAGGATATTGAAAAGATACAGAAGGGAG

Supplementary Table 3: Ion source classification for survey strains and MotB-ASRs. Bacterial species and their ion sources used for the determination of the correlation of mutations at each respective site of MotB ancestral sequences

Strain or ASR Node	Ion Source
node #908	H^+
node #1024	H^+
node #765	H^+
node #981	H^+
node #1459	H^+
node #1501	H^+
node #1457	H^+
node #1246	H^+
node #1170	H^+
node #1239	H^+
node #760	H^+
node #759	H^+
node #758	H^+
MotB *Escherichia coli* strain K12 MOTB ECOLI	H^+
MotB *Salmonella typhimurium* strain LT2 MOTB SALTY	H^+
MotB *Pseudomonas aeruginosa* strain ATCC 15692 Q9HUL2 PSEAE	H^+
MotB *Bacillus subtilis strain* 168 MOTB BACSU	H^+
MotB *Streptococcus pneumoniae* A0A0T8PK69 STREE	H^+
Bacillus licheniformis strain ATCC 14580 Q65KJ0 BACLD	H^+
MotB *Streptococcus pneumoniae* A0A0E8TCW6 STREE	H^+
MotB *Helicobacter pylori* strain ATCC 700392 MOTB HELPY	H^+
MotS *Oceanobacillus iheyensis* A0A2P1WLE1 9BACI	Na^+
MotS *Bacillus alcalophilus* G9I2I5 BACAO	Na^+
MotS *Bacillus subtilis* subsp natto BEST195 BAI864791	Na^+
MotS *Bacillus licheniformis* A0A1Q9FXY5 BACLI	Na^+
MotD *Pseudomonas aeruginosa* strain ATCC 15692 G3XD90 PSEAE	H^+
MotB *Desulfovibrio magneticus* strain ATCC 700980 C4XPD2 DESMR	H^+
PomB *Vibrio alginolyticus* O06874 VIBAL	Na^+
PomB *Vibrio cholerae* serotype O1 strain ATCC 39315 Q9KTK9 VIBCH	Na^+
MotB *Aliivibrio fischeri* KLU777421	Na^+
PomB *Shewanella oneidensis* MR 1 NP 7171461	Na^+
MotB *Aquifex aeolicus* strain VF5 O67121 AQUAE	Na^+

Supplementary Table 4. Pairwise correlation for specific residues with ion source. Significance of the pairwise correlation of residue with phenotype analysed by Fisher's Exact Test

E. coli MotB residue position	Original 19-species subset Pr(>|z|)	Original 19-species subset with our 13 ASR nodes Pr(>|z|)
30	0.0034	0.014
31	0.25	0.031
35	0.14	0.022
36	0.21	0.00082
37	N/A	0.013
38	1	0.12
40	0.033	0.15
43	0.00041	2.8E-05
44	0.21	0.012
48	N/A	1

Supplementary Datasets:
Phylogeny, sequence alignment, and PAML output files for MotB sequence reconstruction are available for download from: https://github.com/phatmattbaker
